# Treatment of Typhoid Fever

**Published:** 1898-12

**Authors:** T. M. Wilson

**Affiliations:** Thornton, Texas


For Texas Medical Journal.

### Treatment of Typhoid Fever.

BY T. M. WILSON, M. D., THORNTON, TEXAS.

**Read at the last meeting of the Texas State Medical Association, at Houston, Texas, April, 1898.**

In presenting this paper I do not think it necessary to discuss at length the etiology, symptomatology and pathology of typhoid fever. I believe it is generally admitted that the disease is an enteric affection, being produced by improper hygiene; such as bad sewerage, contaminated food, and especially impure drinking water. Bacteriologists and pathologists claim that in this water there are certain micrococci or living germs, that when taken into the alimentary canal, find a lodgent in the glands of the bowel or intestine, where they soon multiply to such an extent that the whole system becomes so charged that a fever, which has certain peculiar symptoms, is produced. This fever if not complicated with some other affection, is usually preceded by a dull stupor and more or less headache, which may continue several days before there is any perceptible rise in the temperature. The tongue usually becomes coated with an ash colored fur, while its margins are clean and more or less red. The abdomen generally tender and more or less distended. This last symptom usually continues until convalescence is fully established. We are all aware that typhoid fever generally continues from twenty to forty and sometimes ninety days. The maxim temperature, 103 degrees to 104½ degrees F., is generally reached within the first ten days, and may continue with morning and evening exacerbations for twenty or more days, then slowly declines, especially in favorable cases. Of course, typhoid, like many other fevers, is often complicated with other affections; the most common of which is malaria. Then instead of the usual symptoms at the onset, the patient may have a chill, followed by high temperature, or the high temperature without the chill, which may continue, especially during the evening, for four or five days, then fall two degrees or three degrees and take on a regular form of typhoid. So we often have to wait several days before we can be positive of our diagnosis. But we need lose no time in the treatment, for what is usually good for the one is good for the other. The first thing I do when called to see a patient, is to give a course of calomel in broken doses. If the bowels are loose, I add to each dose bismuth, chalk, soda, and Dover's powder, to be followed in a short time by quinine, which I give in doses of two to four grains every four to six hours, and continue as long as there is fever; I also repeat the calomel two to three times a week as long as there is fever, but do not give enough to weaken the patient. When there is very high fever I bathe the head with cold water, but not the body and extremities, because that is too depressing. Such bathing may be admissible if we have trained nurses; especially in hospital practice. I never use any coal tar salts or their derivatives to reduce fever; they are too depressing. I do sometimes use aconite and gelsemium combined with digitalis so as to counteract their depressing effect. I do this only when the patient objects to cold water on the head, or to having the body and extremities sponged with equal parts of tepid water and vinegar. I sponge the patient's body from one to three times daily with vinegar and water through the entire fever stage; as it seems to soothe or quiet, as well as to reduce the temperature. Should there be much delirium and insomnia, I give bromide of potash and chloral; sometimes sulphuric ether hypodermically. For tenderness of the bowels, I use hot fomentations or poultices containing a little turpentine. If the tongue is dry and crusted, give a little turpentine per mouth; I also use the turpentine if there is the slightest intestinal hemorrhage. Should there be considerable hemorrhage, I use opiates and sugar of lead, and if the patient becomes prostrated from the hemorrhage, give digitalis and nux vomica or strychnia, besides have the patient to drink all the water that can be borne on the stomach, so as to supply the blood-vessels with serum enough for the arterial muscles to help the heart force the blood on its rounds. If there is nausea have the water as hot as can be borne. For vomiting at other times, peppermint, hot starch water, or any of the other anti-emetics usually suffice. If sweating should become too profuse in the latter stages of the disease, atropine is indicated. If there should be persistent diarrhœa, bismuth, chalk, and Dover's powder usually give relief; but if they fail, then opium should be used. However, the patient will get along better if the bowels are kept so as to get two or three actions a day. In all fevers I let my patients drink all the water they desire, if it does not nauseate. Any complications that may arise during the course of the disease I treat with the usual remedies. For the past eight years, as an internal antiseptic and germicide, I have used the following prescription with almost unvaring success:

℞

Ammonia Nitrate............................ʒiv to iv

Fowler's Solution of Arsenic............ʒi

Aquæ dest...................................℥iv

M. S. One teaspoonful in water every three or four hours in daytime and four at night.

I commence this treatment as soon as I suspect my patient has typhoid fever, and continue it till five or six days after the fever has disappeared. Sometimes it seems to nauseate a little; then give it in hot starch water. Since I have adopted this latter treatment, the febrile stage seldom continues longer than ten to fifteen days from the time I am first called to see the patient. The fever generally begins to decline, after three or four days of this latter treatment, at the rate of one-half degree every twenty-fours. In the later stages of the disease the patient often, especially in the early morning hours, needs some good heart or cardiac stimulant, such as nux vomica, digitalis and belladonna, or atropine.

Now as to diet, we should feed enough to sustain the vitality of the patient, but always guard against over-feeding. I am satisfied that many patients succumb to the disease by eating too much, or eating indigestible food. More relapses are caused by over-feeding in tphyoid than from any other cause. It is no uncommon thing, on visiting our patients to find an unexpected rise of temperature caused by something the patient has eaten. In such cases, I immediately give a dose of calomel. I usually restrict the diet to buttermilk and malted milk; of course there are other good invalid foods. To any of them I generally add some of the pepsine preparations. Some tonic and aid to digestion should be kept up two or three weeks after the fever has disappeared. We cannot be too careful in guarding against filth or foul air in or about the sick room. If we have the least suspicion that the water supply is contaminated, we should insist on a change, and if that is inconvenient, then insist on boiling all the water used for drinking purposes.

Now, then, without desiring to be dogmatic, I have given only a brief outline of the treatment which I have been using for the past eight years, a treatment that I believe, were it adopted by the profession generally, would tend very much to relieve us of our embarrassing attitude in the treatment of typhoid fever.

---

THORNTON, TEXAS, October 31st, 1898.

*Gentlemen, Readers of the Texas Medical Journal:*

Why the above paper was rejected by the publishing committee, I leave you to surmise. I know that it was not well received by a considerable portion of the members present, and especially by one of the publishing committee. For in replying to it he did nothing but poke fun at it. He, with several other members present, seemed to cling tenaciously to the old moss-back theory that all that can be done in typhoid fever is to guide or try to hold down the fever, while nature is left or depended upon to do the rest. In the discussion that followed the reading of the paper, I could not tell whether or not the germ or septic theory of typhoid was admitted by the speakers. One of the publishing committee said that in sterilizing the water used, I would have to boil all the ice used by the patient. Though he lives in Galveston, he has never learned the process of ice manufacture. Does not even know that the water is first converted into steam. Another member said he used anti-febrine or some one of the other coal tar preparations to destroy the fever germs; to which I replied that his remedy was certainly a most powerful germicide, but in all his severe cases he would destroy the patient first, for if there is a more powerful heart depressent used as medicine, especially in the doses usually prescribed, I do not know what it is. In typhiod fever we should always strive to sustain the vitality of the patient. In almost all diseases the wise physician tries to overcome or destroy the cause without impairing the vitality of the patient; then why not use some antiseptic or germicide in typhoid, especially if we believe it is caused by a germ or septic poison. In other words, why not use rational medicine in the treatment of typhoid? fever If a Pasteur or a Koch were to proclaim to the world that he had discovered a germicide or anti-bacillus that would abort or lessen the duration of this most distressing and fatal disease, the medical profession would hail it with delight and exclaim, "Great is Pasteur."

Now, gentlemen, I claim no originality in this discovery, but know that in the hands of a wise and judicious physician it will do just what I claim for it. There are probably other germicides or antiseptics that will do more, but having used it with almost perfect success for eight years, I ask a fair trial of its merits by our profession. The antiseptic theory by Woodbridge, I believe to be correct whether his medicine is a success or not. I have never tried his remedy, but believe in giving the patient all the rest that is consistent with the attending circumstances or surroundings. Now, if your patient has what is commonly denominated typho-malaria, try the same treatment and you will be pleased. You should always use fresh nitrate of ammonia, and not allow it to be exposed to light or air.

T. M. WILSON, M. D.

